# Supplementary material for: TMPRSS2 is a tumor suppressor and its downregulation promotes antitumor immunity and immunotherapy response in lung adenocarcinoma
Source: Respir Res. 2024 Jun 11;25:238. doi: 10.1186/s12931-024-02870-7 (PMC11167788; doi:10.1186/s12931-024-02870-7)

Supplementary Figure S1

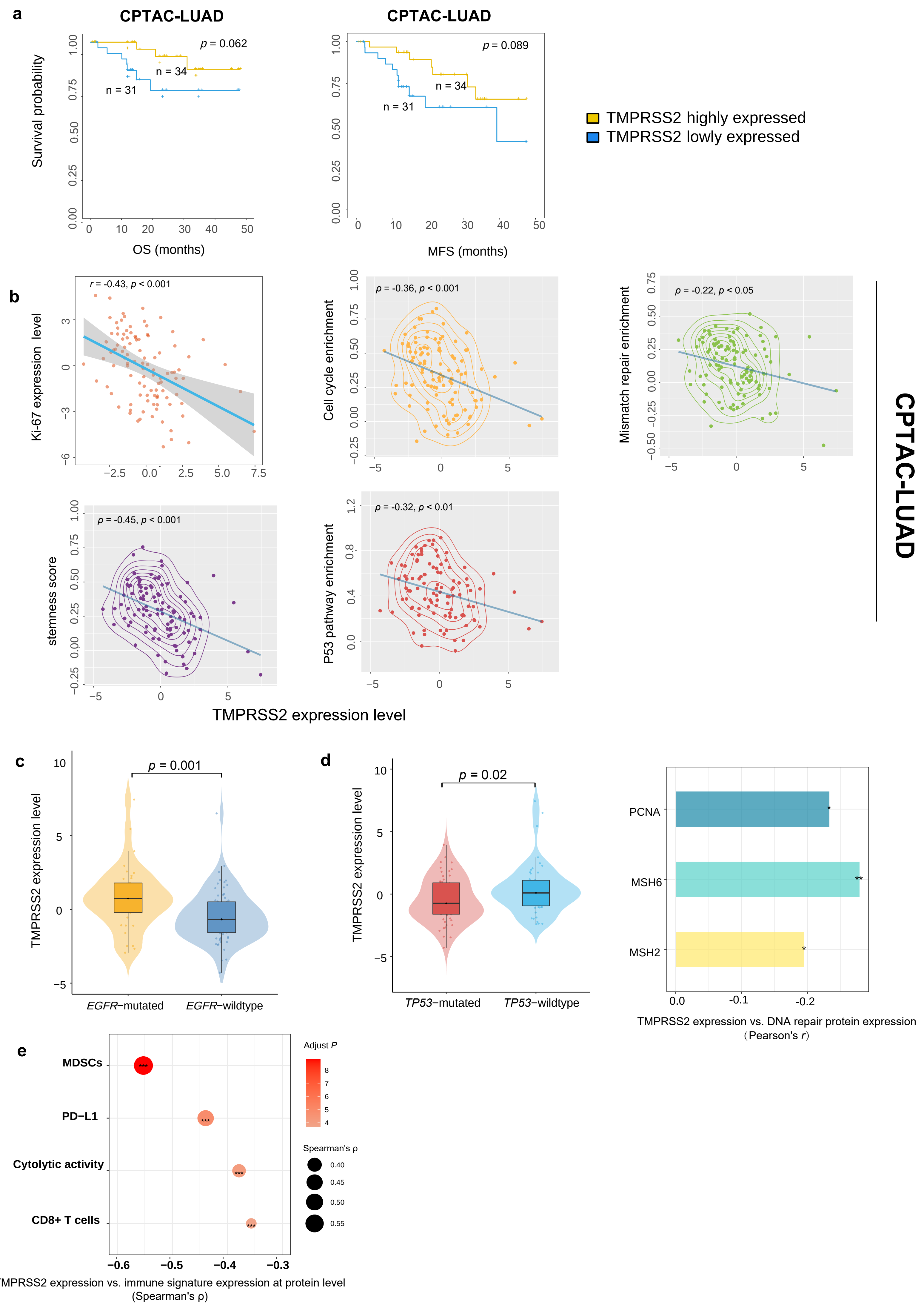

Supplement: Supplementary file 2 — Additional file 2: Figure S1. Validation of the mRNA-based findings at the protein level in CPTAC-LUAD. (a) Kaplan-Meier survival curves showing that LUAD patients with lower TMPRSS2 expression levels (bottom third) have worse OS and MFS than those with higher TMPRSS2 expression levels (upper third). The log-rank test p values are shown. OS, overall survival. MFS, metastasis-free survival. (b) The expression of TMPRSS2 correlates inversely with Ki-67 expression, the enrichment of the cell cycle, mismatch repair, and p53 signaling pathways and the stemness signature in LUAD. (c) TMPRSS2 is more highly expressed in EGFR-wildtype than in EGFR-mutated LUADs. (d) TMPRSS2 is more lowly expressed in TP53-wildtype than in TP53-mutated LUADs and shows negative expression correlations with DR-associated proteins (MSH2, MSH6, and PCNA) in LUAD. (e) TMPRSS2 expression correlates inversely with the enrichment of CD8+ T cells, cytolytic activity, PD-L1, and MDSCs. The Pearson or Spearman correlation coefficients and p values are shown in (b, d, e). * p < 0.05, ** p < 0.01, *** p < 0.001, ns p ≥ 0.05. [file 12931_2024_2870_MOESM2_ESM.pdf]
