# Supplementary figures and images for: TMPRSS2 is a tumor suppressor and its downregulation promotes antitumor immunity and immunotherapy response in lung adenocarcinoma
Source: Respir Res. 2024 Jun 11;25:238. doi: 10.1186/s12931-024-02870-7 (PMC11167788; doi:10.1186/s12931-024-02870-7)

Supplementary Figure S2

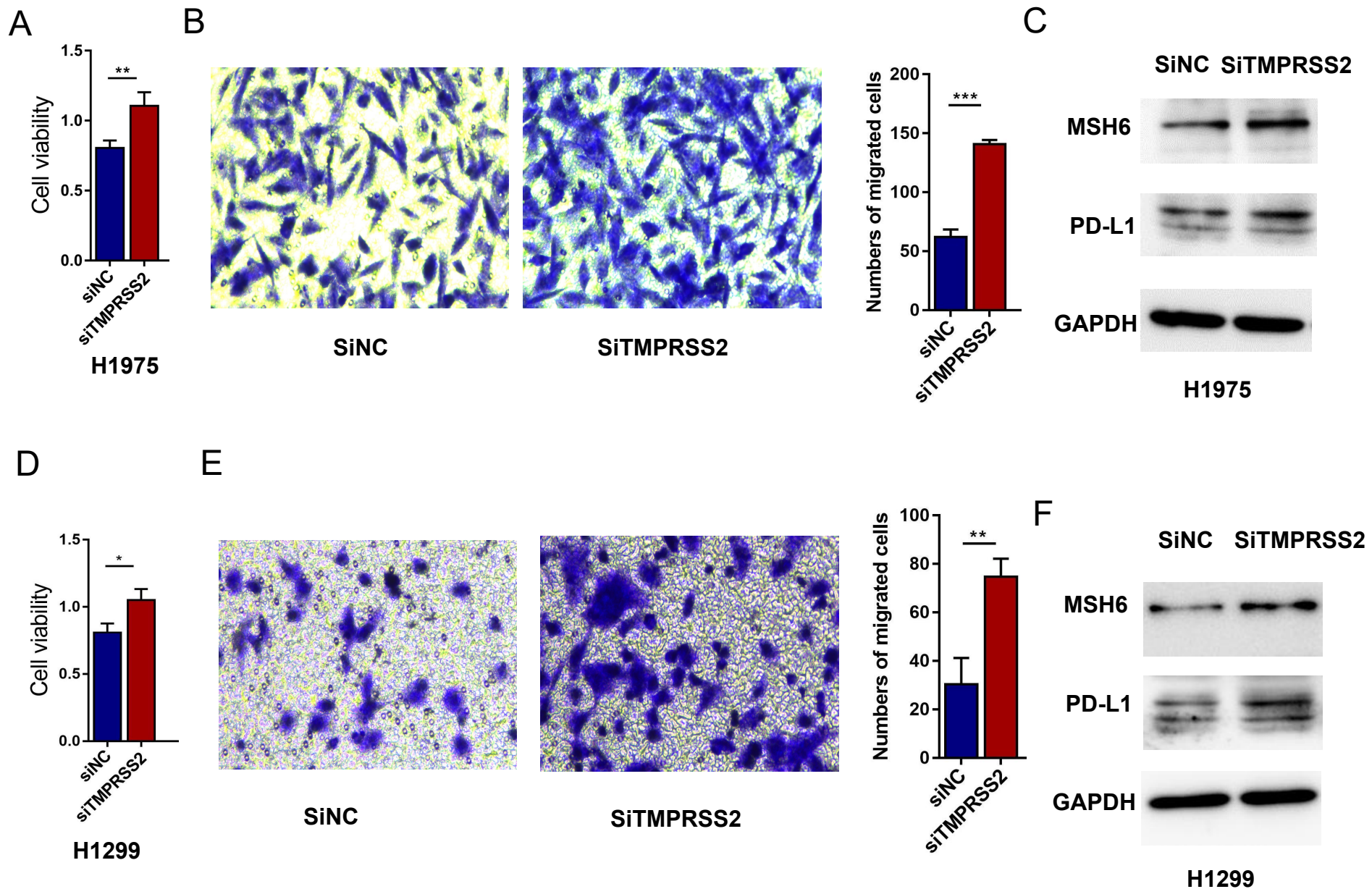

Supplement: Supplementary file 3 — Additional file 3: Figure S2. TMPRSS2 knockdown markedly promoted proliferative and invasive abilities in another two lung adenocarcinoma cells. (A&B). TMPRSS2 knockdown markedly promoted proliferative and invasive abilities of H1975 cells. C. TMPRSS2 knockdown increased MSH6 and PD-L1 expression in H1975 cells. (D&E). TMPRSS2 knockdown markedly promoted proliferative and invasive abilities of H1299 cells. F. TMPRSS2 knockdown increased MSH6 and PD-L1 expression in H1299 cells. [file 12931_2024_2870_MOESM3_ESM.pdf]

Supplementary Figure S1

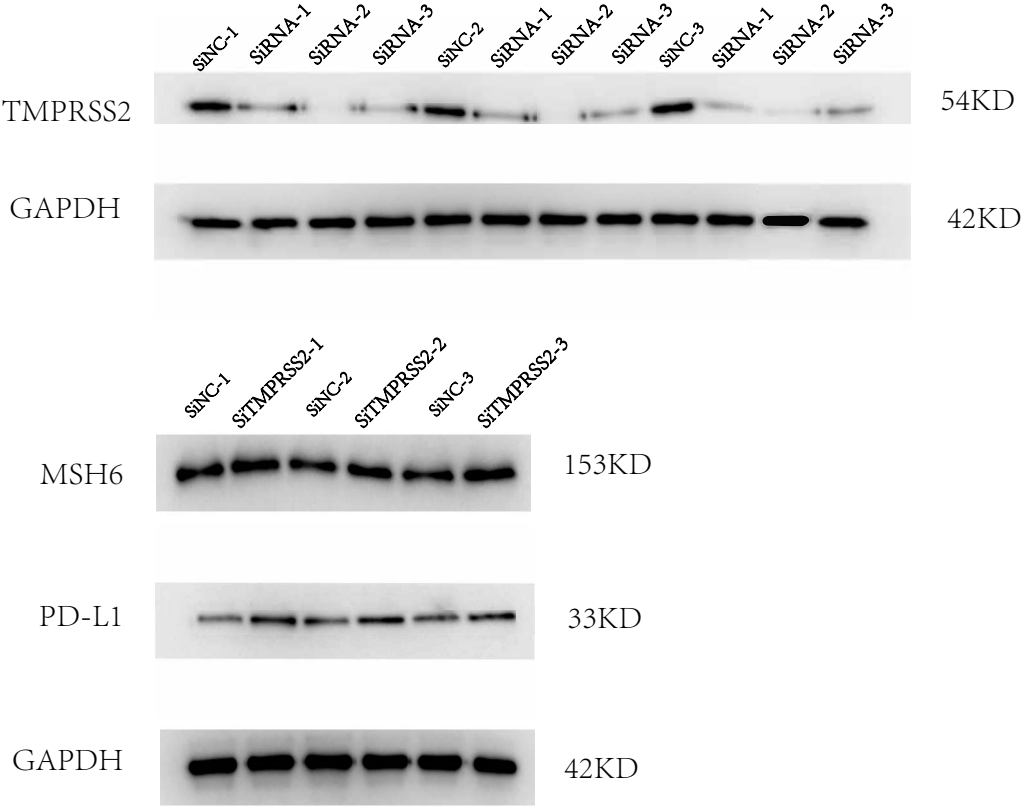

Supplement: Supplementary file 4 — Additional file 4: Figure S3. Full uncropped Gels and Blots images. [file 12931_2024_2870_MOESM4_ESM.pdf]
